# Chikungunya Virus Infection, Brazzaville, Republic of Congo, 2011

**DOI:** 10.3201/eid1909.130451

**Published:** 2013-09

**Authors:** Jean-Vivien Mombouli, Patrick Bitsindou, Darrel O.A. Elion, Allen Grolla, Heinz Feldmann, Fabien R. Niama, Henri-Joseph Parra, Vincent J. Munster

**Affiliations:** Laboratoire National de Santé Publique, Brazzaville, Republic of Congo (J.-V. Mombouli, D.O.A. Elion; F.R. Niama; H.-J. Parra);; Université Marien Ngouabi, Brazzaville (J.-V. Mombouli, F.R. Niama);; Délégation Générale à la Recherche Scientifique et Technologique, Brazzaville (P. Bitsindou);; Public Health Agency of Canada, Winnipeg, Manitoba, Canada (A. Grolla);; National Institutes of Health, Hamilton, Montana, USA (H. Feldmann, V.J. Munster)

**Keywords:** chikungunya virus, viruses, Republic of Congo, Aedes albopictus, Aedes aegypti, Brazzaville, mosquitoes, outbreaks

**To the Editor:** Chikungunya virus (CHIKV) is a positive single-stranded RNA virus within the genus *Alphavirus*, family *Togaviridae* (*1*–*3*). In humans, CHIKV infection has a rapid onset and is typically cleared in 5–7 days. Following transmission, CHIKV replicates in the skin and then disseminates to the liver and joints. The incubation period for CHIKV infection is 2–4 days. Signs and symptoms of infection include high fever, rigors, headache, photophobia, and a petechial or maculopapular rash, and most infected persons also report severe joint pain (*2*–*4*). CHIKV is transmitted through the bite of infected *Aedes aegypti* and *Ae. albopictus* mosquitoes. The current reemergence of CHIKV seems to be related to 1) CHIKV host switching from *Ae. aegypti* to *Ae. albopictus* mosquitoes, while retaining *Ae. aegypti* mosquitoes as a vector, and 2) introduction of *Ae. albopictus* mosquitoes, which were originally from Southeast Asia, into new areas of the world, including Africa. Severe epidemics of CHIKV infection have occurred in countries in the Indian Ocean region and Africa (*1*–*3*). We report an outbreak of CHIKV infection in Brazzaville, Republic of Congo, that was associated with the mosquito vectors *Ae. albopictus* and *Ae. aegypti*.

In June 2011, reports were received of an outbreak of a new drug-resistant form of malaria in southwest Brazzaville. Diagnostic capacity for detecting arboviruses, including CHIKV, by using molecular diagnostic assays was immediately implemented at the Laboratoire National de Santé Publique in Brazzaville. Blood samples were collected from 23 suspected case-patients, and serum samples were obtained and stored at 4°C. RNA from patients’ serum samples was extracted by using the QIAamp Viral RNA Kit (QIAGEN, Valencia, CA, USA) according to the manufacturer’s instructions. To determine if CHIKV, yellow fever virus, dengue virus, or malarial parasites were present in the serum samples, we performed quantitative reverse transcription PCR with Lightcycler 480 RNA Master Hydrolysis Probes (Roche Diagnostics, Mannheim, Germany) on the SmartCycler platform (Cepheid, Sunnyvale, CA, USA) (*5*–*7*). A cycle threshold (C_t_) cutoff value of <40 was considered positive. Primary testing focused on CHIKV, and genomic RNA was detected in 21 of 23 patient serum samples (C_t_ 17.55–37.91) (Table). Neither yellow fever nor dengue virus genomic RNA was detected in any patient samples. However, malaria parasite genetic material was detected in the 2 samples that were negative for CHIKV (Table).

**Table:** Detection of virus genomic RNA in patient serum samples, Brazzaville, Republic of Congo, June 2011*

Patient sample ID	Virus, C_t_			Malaria C_t_
	Chikungunya	Yellow fever	Dengue	
1†	25.62	>40	>40	>40
2	>40	>40	>40	32.33
3	17.55	>40	>40	>40
4	37.91	>40	>40	>40
5	21.36	>40	>40	>40
6	21.00	>40	>40	>40
7	20.26	>40	>40	>40
8	19.78	>40	>40	>40
9	>40	>40	>40	33.71
10	25.77	>40	>40	>40
11‡	21.20	>40	>40	>40
12	22.98	>40	>40	>40
13	29.97	>40	>40	>40
14	23.50	>40	>40	>40
15§	31.97	>40	>40	>40
16	31.44	>40	>40	>40
17	33.54	>40	>40	>40
18	31.83	>40	>40	>40
19	34.63	>40	>40	>40
20	34.90	>40	>40	>40
21	33.46	>40	>40	>40
22	29.18	>40	>40	>40
23	33.16	>40	>40	>40

*Except as otherwise indicated, all samples were collected on June 27, 2011. ID, identification; A C_t_ cutoff value of <40 was considered positive. C_t_, cycle threshold. †Collected on June 23, 2011. ‡Collected on June 24, 2011. §Collected on June 27, 2011.

To investigate the involvement and distribution of potential vector mosquito species, we initiated an entomological study in the Makélékélé and Mfilou districts of Brazzaville, the 2 areas associated with the CHIKV outbreak. Mosquitoes were collected at various places in the 2 districts during different hours of the day (Technical Appendix Table). Mosquitoes were separated by species, *Ae. aegypti* or *Ae. albopictus*, into 13 different pools. The pools were homogenized in lysis buffer (Buffer AVL; QIAGEN) by using sterile disposable tissue grinders, and RNA was extracted. We then analyzed the samples for the presence of CHIKV genomic RNA by following the procedures described above for the serum samples. All 13 mosquito pools were positive for CHIKV genomic RNA (C_t_ 23.5–34.63) (Technical Appendix Table).

Our study in Brazzaville identified CHIKV as the causative agent for the 2011 outbreak of febrile illness characterized by severe joint pain. In the affected Brazzaville communities, the illness had been named robot malaria because of its effects on posture and locomotion, effects that are typical of CHIKV infection. We also found that during the outbreak, CHIKV was present in *Ae. aegypti* and *Ae. albopictus* mosquitoes, suggesting that these mosquitoes played a role in dissemination and spread of the virus. These findings are in line with the increasing distribution of CHIKV and its vector, *Ae. albopictus* mosquitoes, in Africa. 

Of note, the outbreak in the Republic of Congo seems to have been associated with *Ae. aegypti* and *Ae. albopictus* mosquitoes, whereas outbreaks of CHIKV infection in neighboring Gabon are predominantly associated with *Ae. albopictus* mosquitoes (*8*,*9*). Our study confirms the suspected presence of *Ae. albopictus* mosquitoes in the Republic of Congo. The identification of *Ae. albopictus* mosquitoes as a vector for CHIKV in remote areas in Gabon indicates that similar remote areas in the Republic of Congo could also be at risk during future outbreaks of CHIKV infection (*8*).

The availability of diagnostic capacity for arbovirus detection in sub-Saharan Africa is urgently needed for the deployment of potential intervention strategies. Improved surveillance and rapid identification of novel arbovirus–associated outbreaks would strengthen the potential for recognition of arbovirus-related diseases by health care providers and public health officials. In the absence of CHIKV-specific prophylactic or therapeutic control measures, the only current mitigation strategies are based on community outreach, which provides early alerts regarding outbreaks, and on control of the mosquito vectors (*10*).

Technical Appendix Detection of virus genomic RNA in mosquito pools, Brazzaville, Republic of Congo, June 2011*
