# Supplementary material for: Chikungunya Virus Infection, Brazzaville, Republic of Congo, 2011
Source: Emerg Infect Dis. 2013 Sep;19(9):1542–3. doi: 10.3201/eid1909.130451 (PMC3810930; doi:10.3201/eid1909.130451)
Supplement: Technical Appendix — Detection of virus genomic RNA in mosquito pools, Brazzaville, Republic of Congo, June 2011* [file 13-0451-Techapp-s1.pdf]

Article DOI: http://dx.doi.org/10.3201/eid1909.120451

Chikungunya Virus Infection, Brazzaville, Republic of Congo, 2011

Technical Appendix

Technical Appendix Table. Detection of virus genomic RNA in mosquito pools, Brazzaville, Republic of Congo, June 2011*

Mosquito pool ID	Time of day	Species	Collection location in Brazzaville	Chikungunya virus, C_t
1	9–10 AM	*Aedes albopictus*	Makélékélé, Djoué	29.97
2	9 AM–3 PM	*Ae. albopictus*	Mfilou	23.5
3	9 AM–12 PM	*Ae. aegypti*	Makélékélé, Djoué	31.97
4	5–6 PM	*Ae. aegypti*	Makélékélé, Djoué	31.44
5	5–6 PM	*Ae. albopictus*	Makélékélé, Djoué	33.54
6	5–6 PM	*Ae. albopictus*	Makélékélé, Djournouna	31.83
7	5–6 PM	*Ae. aegypti*	Makélékélé, Djoué	34.63
8	5–6 PM	*Ae. albopictus*	Makélékélé, Djoué	34.9
9	9 AM–12 PM	*Ae. albopictus*	Makélékélé, Orstom	33.46
10	2–6 PM	*Ae. aegypti*	Makélékélé, Djoué	29.18
11	2–6 PM	*Ae. aegypti*	Makélékélé, Madibou	33.16
12	9 AM–12 PM	*Ae. aegypti*	Makélékélé, Djoué	31.2
13	9 AM–12 PM	*Ae. albopictus*	Makélékélé, Djoué	32.82

*All samples were collected on June 27, 2011. ID, identification; A C_t cutoff value of <40 was considered positive. C_t, cycle threshold.
